# An explainable machine learning model for predicting chronic coronary disease and identifying valuable text features

**DOI:** 10.3389/fcvm.2025.1559831

**Published:** 2025-09-22

**Authors:** Weipeng Gan, Peipei Wang, Xiangrong Xie, Lingfei Yang, Dasheng Lu, Sheng Ye, Mingquan Ye

**Affiliations:** ^1^ Department of Cardiovascular Medicine, The Second Affiliated Hospital of Wannan Medical College, Wuhu, Anhui, China; ^2^ School of Medical Information, Wannan Medical College, Wuhu, Anhui, China; ^3^ Department of Cardiovascular Medicine, The First Affiliated Hospital of Wannan Medical College, Wuhu, Anhui, China

**Keywords:** chronic coronary disease, pre-test probability, text mining, machine learning, early diagnosis

## Abstract

**Background:**

Chronic Coronary Disease (CCD) is a leading global cause of morbidity and mortality. Existing Pre-test Probability (PTP) models mainly rely on in-hospital data and clinician judgment. This study aims to construct machine learning (ML) models for predicting CCD by using easily accessible text data and baseline characteristics, and to evaluate the contribution of text data to the diagnostic model.

**Methods:**

The chief complaints, present illness, past medical history and vital signs of the patients from the internal medicine departments of the First Affiliated Hospital and the Second Affiliated Hospital of Wannan Medical College were gathered. The text data of the research subjects were structured by using text mining technology. A customized “stop words” list and “custom dictionary” for cardiovascular medicine were created to optimize the processing of text data. Then, ML algorithms were employed to establish CCD prediction models. Finally, the Shapley additive explanation (SHAP) algorithm was used to interpret the models.

**Results:**

We enrolled a total of 21,855 patients in this study, with 7,449 in the CCD group and 14,406 in the non-CCD group. Patients in the CCD group were generally older and had a higher male proportion. After conducting feature engineering, we successfully constructed a Random Forest model. The model achieved an area under the ROC curve (AUC) of 0.93 (95% CI, 0.93–0.94), demonstrating excellent performance in horizontal comparisons. Using the SHAP algorithm, valuable text features like “chest pain”, “chest tightness” and structured features such as age, which are crucial for CCD judgment, were identified. Additionally, an illustration of how these features influenced the model's decision-making process was provided.

**Conclusion:**

Clinicians can leverage text data to construct a prediction model for CCD and apply the SHAP approach to pinpoint valuable text features and elucidate the model's decision-making mechanism.

## 1 Introduction

Chronic Coronary Disease (CCD) is one of the leading causes of morbidity and mortality globally (1). Accurately predicting CCD remains a significant challenge in clinical practice (2).

The assessment of Pre-test Probability (PTP) plays a pivotal role in the management of this disease, a role explicitly integrated into the entire diagnostic evaluation process by the 2023 Guideline for the Management of CCD (1). The guideline emphasizes that PTP plays a decisive role in guiding the selection of non-invasive tests and risk stratification. It is recommended to conduct a preliminary PTP assessment by comprehensively considering the patient's age, gender, typical symptoms, and cardiovascular risk factors such as diabetes and smoking history. In clinical practice, this assessment hinges on clinicians’ professional judgment, medical knowledge, and thorough symptom evaluation—and directly shapes subsequent diagnostic strategies.

In CCD prediction, prior studies have developed effective models using diverse variables. Reeh J et al. integrated basic variables (angina type, gender, age) and clinical risk factors (diabetes, family history of coronary heart disease, dyslipidemia), achieving a C-statistic of 0.88 to enhance diagnostic efficiency (3). Similarly, Mirjalili SR et al. established a machine learning model with biochemical indicators (lipids, blood glucose), successfully identifying high-risk CHD individuals with 87.5% accuracy (4). In addition, the 2019 European Society of Cardiology (ESC) guideline also proposed a coronary heart disease prediction model based on coronary computed tomography angiography (5). However, current PTP models still have certain limitations. These models predominantly rely on in-hospital imaging data, laboratory test parameters, and subjective assessments of symptom characteristics by clinicians (1, 6, 7). This restricts the application of these models in scenarios such as pre-hospital emergency care and primary healthcare.

A large amount of textual information about patients’ medical conditions and treatment experiences is generated when they seek medical treatment, which is crucial for diagnosis (8, 9). Experienced doctors can make accurate preliminary diagnoses based on patients’ symptoms and signs, and then verify these diagnoses through clinical laboratory tests and imaging results (10). However, these data are often unstructured. Due to their large volume, variable structure, difficulty in information extraction, and the lack of a unified evaluation standard, they pose many challenges to clinical research. Therefore, it is difficult for clinical researchers to fully utilize this resource to develop disease prediction models and conduct risk assessments (11).

In recent years, with the rapid development of artificial intelligence technology, the applications of text mining and machine learning (ML) in the medical field have significantly increased (12, 13).

The combination of text mining and ML technology can analyze the text data from patients’ subjective descriptions, clinical records, and medical histories, extract key information and features, and provide valuable insights for disease prediction. For example, Zhu E et al. proposed a unified framework that can efficiently annotate and extract key information from Chinese clinical texts (14). Tauscher JS et al. developed an automatic cognitive distortion detection model, which is capable of identifying potential communication problems by analyzing the text of doctor-patient conversations, thus providing objective support for the diagnosis and treatment of mental illnesses (15). Bergman E's team developed a BERT-based natural language processing system that has achieved an accuracy close to the human level in the classification of adverse drug reaction reports, leading to a significant improvement in the efficiency of drug safety monitoring (16).

Existing models rely on high-cost and hard-to-obtain data, whereas text data, with its advantages of easy accessibility and inclusion of subjective symptoms, can address this limitation. Thus, this study is committed to exploring its role in evaluating the PTP of patients with CCD. We have constructed a CCD identification model by incorporating text data and easily accessible structured data. Moreover, this study conducts an interpretive analysis to explain the model's decision-making process. The ultimate aim is to establish a cost-effective model for clinical practice, facilitating the assessment of CCD patients’ PTP.

## 2 Materials and methods

### 2.1 Study design and population

This was a dual-center, observational, and retrospective study. The subjects of this research were adult patients who sought medical treatment at the Department of Internal Medicine of the First Affiliated Hospital and the Second Affiliated Hospital of Wannan Medical College. These patients presented with symptoms related to CCD as their chief complaints from January 1, 2022, to December 31, 2024. To mitigate the potential paucity of CCD patient data, the cardiology department data were extended from January 1, 2019, to December 31, 2024. Referring to previous research findings, the symptoms related to CCD include: chest pain, dyspnea, retrosternal discomfort, fatigue, palpitations, dizziness, nausea, discomfort under the xiphoid process, upper abdominal discomfort, neck discomfort, radiating pain to the shoulder, radiating pain to the mandible, and arm pain (1). Exclusion criteria include: (1) Emergency admissions or transferred patients; (2) Cases with unclear diagnosis at discharge; (3) Cases involving heart failure or cardiac function decline, potentially resulting from various cardiovascular diseases (CVDs) and not serving as the primary diagnosis, were excluded.

This study was approved by the Ethics Committee of the Second Affiliated Hospital of Wannan Medical College (Approval: WYEFYLS2025023). Given the retrospective design of this study and the absence of intervention measures, the Ethics Committee waived the requirement for informed consent.

### 2.2 Data processing and feature engineering

#### 2.2.1 Data cleaning

This research used the electronic medical record (EMR) system to extract key data, including admission route, number of hospitalizations, length of stay, and primary diagnoses. To ensure data uniqueness and consistency, patient identification numbers were used as unique identifiers. Primary diagnoses were used to determine whether a condition pertained to CCD. The modeling data included chief complaints, current medical history, past medical history, smoking and drinking histories, vital signs (weight, temperature, blood pressure, pulse), and demographic information (age, gender).

Structured data with obvious errors were manually rectified, and the K-nearest neighbors (KNN) algorithm was applied to impute missing values (k = 3). This approach accurately mirrored the actual relationships among data points and optimized model performance. Moreover, labeling information was removed from the text data to preclude interference in disease classification.

#### 2.2.2 Feature engineering

To convert high-dimensional textual data into a numerical format suitable for modeling, we employed the Jieba Chinese segmentation tool to decompose the text into independent vocabulary units. Subsequently, the TF-IDF (Term Frequency-Inverse Document Frequency) method was adopted to vectorize the segmented features. This approach considered both the term frequency and the inverse document frequency, effectively quantifying the significance of vocabulary within the text.

During the word-segmentation phase, a custom dictionary was introduced to ensure the integrity of medical terms, and stop words were removed to reduce data noise. The custom dictionary and stop-word list were developed based on the medical specialized stop-word dictionary of Harbin Medical University and the THU Open Chinese Lexicon (THUOCL) medical term list from Tsinghua University, which are freely accessible at https://github.com/goto456/stopwords and http://thuocl.thunlp.org/ (17, 18). By integrating medical professional knowledge and regular expressions, and leveraging data from the EMR database of the Affiliated Hospital of Wannan Medical College, we extracted and summarized a unique “custom dictionary” and “stop-words” specific to the Department of Cardiology.

The calculation of TF-IDF refers to the following formula:

$$w_{x,y}=tf_{x,y}\times \left(\frac{N}{df_{x}}\right)$$

Where, ***w_x_*_,*y*_** represents the TF-IDF weight of term ***x*** within document ***y***. ***tf_x_*_,*y*_** (Term Frequency): It indicates the frequency of term ***x*** appearing in document ***y***. Usually, it is calculated as the number of times term ***x*** appears in document ***y*** divided by the total number of terms in document ***y***. It reflects the importance of term ***x*** within a single document ***y***. ***df_x_*** (Document Frequency): It refers to the number of documents that contain term *x*. It measures the commonness of term ***x*** across the entire document collection. ***N*** (Total number of documents): It represents the total number of documents in the document collection.

Data were recorded in Excel 2021 and subsequently analyzed using Python 3.11.1 and scikit-learn 1.2.2.

### 2.3 Development of CCD prediction model

In order to mitigate the risk of overfitting and enhance the interpretability of the model, this study adopted the variance selection method in combination with expert opinions to conduct the selection of target features. Considering the medical modeling scenarios and taking into account both statistical significance and stability, the selected features all had a *P*-value < 0.01. For the standardized continuous features, the variance threshold was set at 0.5. The variance threshold for categorical features was chosen as 0.05. For high-dimensional and sparse text features, the variance threshold was set at 0.001. Meanwhile, referring to expert experience and the results of previous studies, the feature variables finally included in the model were determined.

Considering that the data incorporated into the model consisted of categorical features, continuous features, and text features, during the model construction phase, this study compared various machine-learning algorithms. These included linear algorithms (logistic regression), tree-based algorithms (Random Forest, RF), and clustering algorithms (K-means clustering, KNN). To evaluate the performance of the models, the Receiver Operating Characteristic (ROC) curve, precision, recall, accuracy, and F1-score were employed. Subsequently, the model that exhibited a superior area under the ROC curve (AUC) value was chosen as the final prediction model. Additionally, the performance of our model was compared with previously established models in the literature using the AUC to validate its competitive advantage (1, 2, 19, 20).

### 2.4 Model explanation

This study employed the game-theory-based Shapley additive explanation (SHAP) algorithm to decompose model predictions into individual feature contributions, with the formula:

$$\phi_{i}=\underset{S\subseteq N\setminus \{i\}}{\sum }\frac{|S|!(n-|S|-1)!}{n!}[v(S\cup \{i\})-v(S)]$$

Here, $\phi_{i}$ denotes the SHAP value for the i-th feature; *S* is any subset excluding feature *i*; and v(S) represents the model's baseline prediction using only subset S features. The weight term depends on subset size and total feature count n. Calculation involves iterating over all subsets without feature i, computing marginal prediction changes upon its inclusion, and summing these via weighted aggregation to derive each feature's contribution. Notably, the total of all SHAP values equals the gap between the model's prediction and baseline, ensuring interpretive consistency.

The TreeExplainer tool was used to compute global SHAP values. A summary plot was generated to visualize feature importance and identify key predictive factors. To further explore the model's decision-making logic for individual samples, the SHAP values were calculated using the KernelExplainer tool to provide local interpretations.

## 3 Result

### 3.1 Characteristics of the study population

**Figure 1:**
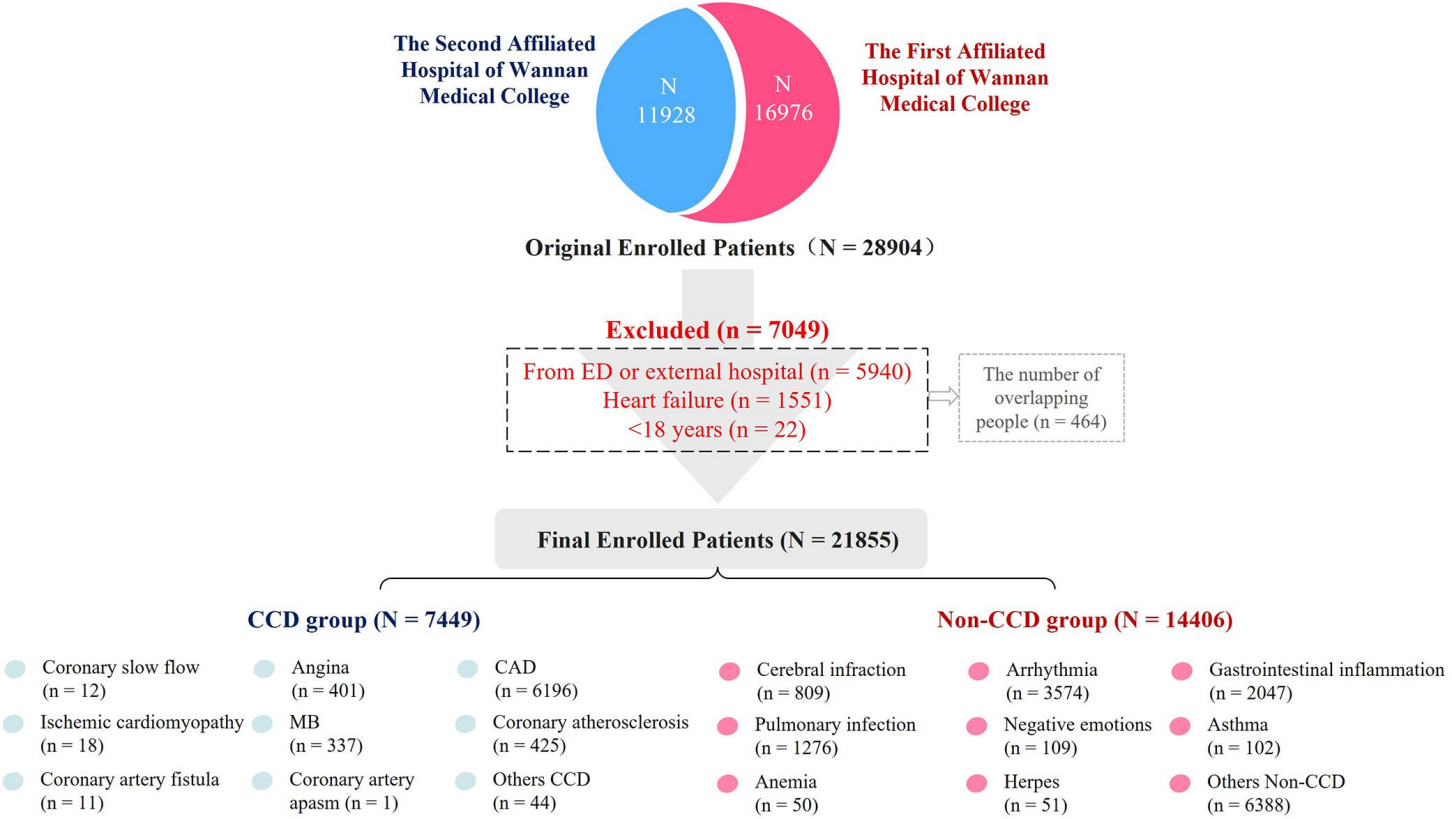
Study flowchart. *CAD, coronary artery disease; MB, myocardial bridge; Others CCD: subacute myocardial infarction, After stent implantation, variant angina pectoris. Others Non-CCD: Pulmonary embolism, pneumothorax, arterial dissection, diabetes mellitus, vertigo syndrome, sleep apnea, congenital heart disease, gallstones, etc.

In total, 21,855 patients with symptoms similar to CCD were admitted to the hospital and enrolled in this study according to the inclusion and exclusion criteria. Among them, 7,449 cases were considered to be admitted due to CCD. In the CCD group, 6,196 cases were clinically diagnosed with coronary heart disease, 401 with angina pectoris, and 337 with coronary myocardial bridges (MB). The remaining 14,406 patients were in the Non-CCD group, including 1,276 cases of pulmonary infectious diseases, 102 cases of asthma, 2,047 cases of digestive system inflammation, and 3,574 cases of arrhythmia, as presented in Figure 1.

As Table 1 shows, the CCD group generally had an older age, a higher male proportion, higher systolic blood pressure, and lower diastolic blood pressure compared to the Non-CCD group. These differences were statistically significant.

**Table 1:** Patient characteristics at baseline.

Features	Non-CCD group	CCD group	*P value*
Gender (Males %)	6,974 (48.41%)	4,149 (55.70%)	<0.01^a^
Smoking (%)	1,583 (10.99%)	1,047 (14.06%)	<0.01^a^
Drinking (%)	1,750 (12.15%)	719 (9.65%)	<0.01^a^
Age	64.00 (54.00, 73.00)	68.00 (58.00, 75.00)	<0.01^a^
Temperature	36.50 (36.30, 36.60)	36.37 (36.30, 36.50)	<0.01^a^
Pulse	78.00 (70.00, 87.00)	76.00 (69.00, 86.00)	<0.01^a^
SBP	134.00 (120.00, 148.00)	135.00 (122.00, 149.00)	<0.01^a^
DBP	78.00 (69.00, 86.00)	76.00 (68.00, 84.00)	<0.01^a^
Weight	63.00 (56.50, 71.00)	64.67 (58.67, 70.67)	<0.01^a^

^a^ CCD, chronic coronary disease; SBP, systolic pressure; DBP, diastolic blood pressure.

### 3.2 Feature selection and model evaluation

**Figure 2:**
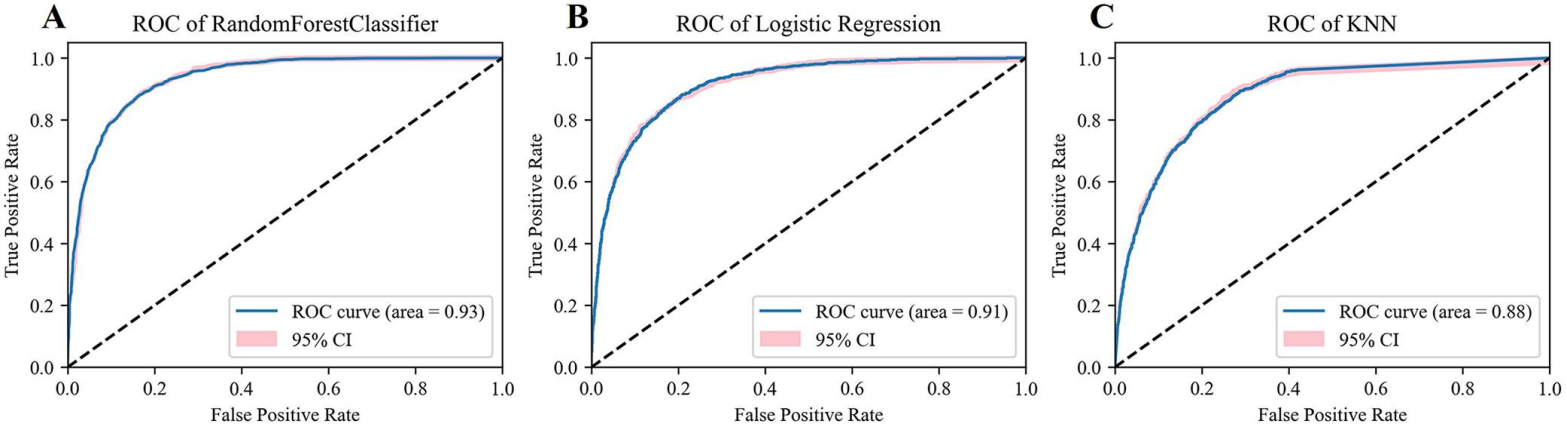
Model evaluation. **(A)** The ROC and AUC of Radom Forest. **(B)** The ROC and AUC of Logistic Regression. **(C)** The ROC and AUC of KNN.

By integrating the variance selection method and expert opinions, this study ultimately identified seven structured features (Gender, Age, Temperature, Pulse, DBP, Smoking, Drinking) along with 129 text features. Since most of these features exhibited non—normal distribution data, we carried out normalization processing on all features. The dataset was then split into a training set (80% of samples) and a validation set (20% of samples) to ensure model training and performance evaluation were conducted on independent data subsets. After normalization, the RF, KNN, and Logistic Regression models were successfully constructed. To optimize the performance of each model, the grid search algorithm was employed in combination with five-fold cross-validation to fine-tune the hyperparameters (For the specific hyperparameter data, the screened feature structure and the data form of the finally constructed text data after TF-IDF processing, please refer to the Supplementary Materials). Subsequently, the Receiver Operating Characteristic (ROC) curves of each model and their 95% confidence intervals were plotted, shown in Figure 2.

Model evaluation results showed that the RF model performed well, achieving an AUC as high as 0.93 (95% CI, 0.93–0.94). In contrast, the AUC of the KNN model was 0.88 (95% CI, 0.87–0.89); the AUC of the Logistic Regression model was 0.91 (95% CI, 0.91–0.92), shown in Figures 2A–C. Further analysis of the classification performance metrics of the RF model revealed that its accuracy, precision, recall, and F1-score (0.86, 0.82, 0.78, 0.80) were superior to those of the Logistic Regression model (0.84, 0.80, 0.73, 0.76) and the KNN model (0.80, 0.76, 0.64, 0.70).

In conclusion, considering various evaluation metrics, the RF model significantly outperformed the KNN and Logistic Regression models in terms of prediction ability and stability, presenting the most excellent model performance. Therefore, this study selected the RF model for further interpretive analysis.

### 3.3 Horizontal comparison of models

A horizontal comparative analysis of multiple models was meticulously carried out in this study, and the results are shown in Table 2. The data analysis reveals that the CCD model developed in this study demonstrates remarkable diagnostic efficiency. The AUC of this model is 0.93, which is significantly higher than that of the model constructed solely based on basic data (AUC = 0.76) and the deep-learning model based on facial images (AUC = 0.73). However, it lags behind the model that combines different types of information, like clinical electrocardiogram data, imaging test results, and functional assessments like ramp test (AUC = 0.95).

**Table 2:** Horizontal comparison of models.

Model	Method	Data categories	AUC, 95% CI	PTP
Our text model	Random forest	Demographic characteristic, Vital sign, Other clinical data: Text	0.93 (0.93–0.94)	True
ESC 2019 PTP model	Logistic regression	Demographic characteristic, Vital sign, personal history	0.76 (0.75–0.77)	True
Reeh et al. Basic model	Logistic regression	Demographic characteristic, Vital sign,	0.74 (0.73–0.75)	True
Reeh et al. Clinical model	Logistic regression	Demographic characteristic, Vital sign, Medical history	0.76 (0.75–0.76)	True
Hassan ChA ul et al. Hospital-internal Model	Random forest	Demographic characteristic,Vital sign, Medical history, Laboratory test, Imaging examination, Functional examination	0.95 (None)	False
Shen Lin et al. Image model	Neural networks	Demographic characteristic data, Vital sign data, Other clinical data: Facial photo	0.73 (0.70–0.76)	True

### 3.4 Model interpretative analysis

**Figure 3:**
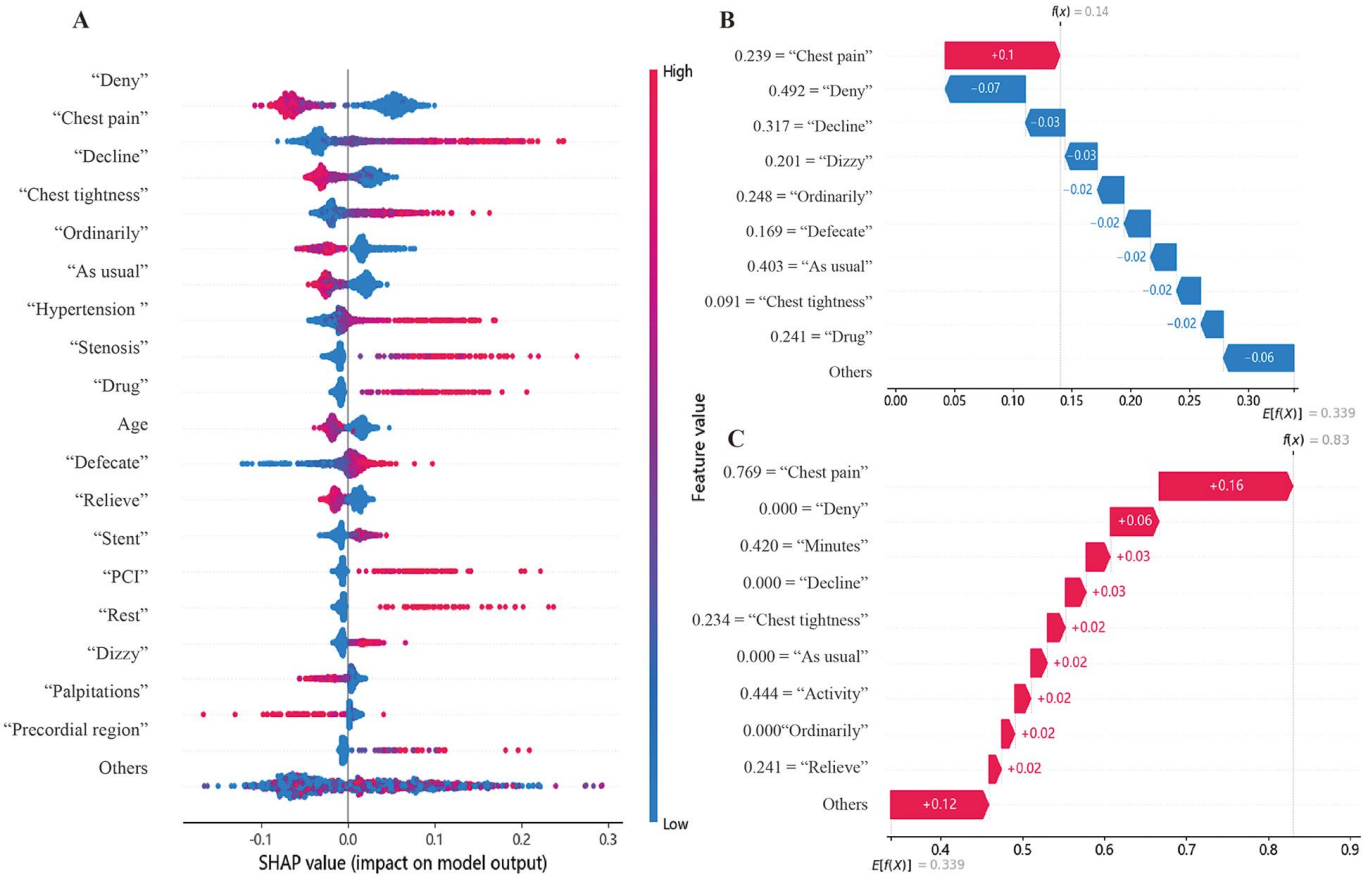
Model interpretative analysis. **(A)** Top Features in SHAP Analysis. In the graph, a point with a redder color indicates a higher value of this feature, while a point with a bluer color indicates a lower value. A point close to the right of the coordinate axis suggests that this feature is more valuable for determining whether a patient has CCD. Meanwhile, a point close to the left of the coordinate axis suggests that this feature is more valuable for determining whether a patient does not have CCD. **(B,C)** Example of SHAP interpretation. The *x*-axis represents important features along with their corresponding values. Features in red have a positive impact on the CCD judgment, while those in blue have a negative impact. f(x) is the final SHAP value for this patient, and E[f**(X)**] is the expected value. When f(x) > E[f**(X)**], a positive judgment is favored.

After successfully constructing a classification model with the RF algorithm, we employed the SHAP algorithm to conduct an interpretive analysis of the model and evaluate the importance of the features. Figure 3A shows the top 20 important features ranked in descending order of the mean absolute SHAP values. Among them, in terms of text variables, “Deny”, “Chest pain”, “Decline”, and “Chest tightness” are among the top-ranked ones. Regarding “Chest pain” and “Chest tightness”, higher TF-IDF values have a greater value for determining the positive diagnosis of CCD. However, for variables such as “Deny” and “Decline”, higher TF-IDF values are more significant for determining the negative diagnosis of CCD. In the structured data, as the value of Age increases, its reference value for diagnosing CCD as positive becomes higher.

This study conducted an interpretation of individual cases based on the local interpretability of SHAP. Figure 3B shows a case where, despite the text information mentioning “Chest pain” (TF-IDF = 0.239), the SHAP values suggested that the risks related to the remaining features were low. This contributes to the model achieving a SHAP value of 0.14, which is lower than the expected value of 0.339. As a result, this drives the model to make a correct negative prediction. In the case shown in Figure 3C, text features like “Chest pain” (TF-IDF = 0.216), “Chest tightness” (TF-IDF = 0.494), and “Activity” (TF-IDF = 0.625) contribute to the model achieving a SHAP value of 0.64, which is higher than the expected value of 0.339. This drives the model to make a correct positive prediction.

## 4 Discussion

This study innovatively utilized easily accessible text data and structured data to construct a cost-effective predictive model for patients with CCD. It has been found that the AUC of this model is as high as 0.93. This model holds potential value in fields such as the identification of cardiovascular diseases in primary healthcare and pre-hospital triage, and the research further highlights the significant role of text data in disease prediction.

Previous clinical studies have also explored integrating text and structured data, focusing on extracting valuable information from complex clinical records to support decision-making. For example, Jonnagaddala J et al. proposed a rule-based risk factor extraction system to identify Framingham heart failure symptoms from clinical records and electronic health records (21). However, their approach solely relied on predefined rules, which led to information gaps beyond these rules and the necessity to reconstruct rules for different problems. In contrast, our study processed text data using algorithms, showing broader applicability. In the face of the complexity of medical texts (16) and the difficulties in processing Chinese texts (22–24), this study optimized the general stop word list and custom dictionary based on the excellent jieba Chinese word segmentation tool (25), medical expertise, and in close accordance with the writing habits of clinicians. As a result, redundant information was successfully removed, and the content valuable for disease diagnosis was retained. Finally, a predictive model was successful constructed, and the decision-making process was demonstrated through explainable machine learning.

When making model comparisons, the machine learning model constructed by Ch Anwar ul Hassa et al. using biochemical indicators, electrocardiograms, and the ramp test showed better predictive performance than our model (19). However, it should be noted that since its data foundation relies on in-hospital diagnostic data, the model outputs are closer to post-test probabilities rather than pre-test probabilities. This characteristic limited the application scenarios of this model. Our study successfully established a CCD predictive model by calculating the TF-IDF values of key words in the text, which can be applied to pre-hospital screening and early diagnosis.

In the model developed in our study, the decision-tree-based ensemble algorithm (RF model) significantly outperforms the KNN and logistic regression models in terms of performance. This advantage may stem from multiple factors. Firstly, tree-based algorithms are more robust to high-dimensional sparse features. The sparse matrices generated by TF-IDF (Term Frequency-Inverse Document Frequency) can trigger the “curse of dimensionality” in KNN. This phenomenon causes the distance metric to fail. In contrast, decision trees can effectively reduce the complexity of the feature space through a recursive feature splitting strategy (26). Secondly, the ability to recognize non-linear patterns is a crucial advantage. In medical texts, there are numerous complex semantic relationships, such as negation modifiers and symptom combinations (e.g., “exertional dyspnea + paroxysmal nocturnal dyspnea”). Decision trees naturally capture feature interactions through hierarchical splitting—unlike linear models like logistic regression, which require manual extraction and matching of such information and ensemble methods, by aggregating predictions from multiple decision trees, further enhance this advantage by resisting overfitting, a problem that plagues single models like KNN or logistic regression, especially with noisy, unevenly distributed medical text data (27).

This study has several limitations. First, the NLP algorithms used are relatively basic. While the random forest approach mitigates, to some extent, the challenge of analyzing the diagnostic value of co-occurring terms (e.g., “chest tightness” and “activity”) for CCD, result interpretation remains heavily reliant on specialists’ judgment. Due to constraints on Chinese medical data resources, there is currently no publicly available Simplified Chinese medical BERT model; however, further enhancements in result accuracy could be achieved through fine-tuning general Chinese BERT models. Moreover, machine learning algorithms have low computational requirements, retaining advantages in terms of cost-effectiveness and accessibility. Second, the professionalism, consistency, and completeness of patients’ symptom descriptions are influenced by physicians’ capabilities and recorders’ subjective judgments, directly limiting text mining accuracy and applicability. Though generative large models lack precision in disease identification, they show promise in data generation and standardization. Existing speech-to-text systems based on such models can convert verbal descriptions into standardized medical texts. Future integration of these technologies with our model to build an integrated “voice collection-text generation-disease identification” system could enable rapid, accurate CCD screening in primary care, easing medical resource pressures. Furthermore, the model was developed using data solely from tertiary hospitals in Wuhu, Anhui Province, China; its applicability across different regions and institution levels requires further validation.

## 5 Conclusion

Our results demonstrate the feasibility of developing a cost-effective prediction model for CCD through the integration of text and structured data. Our model accurately predicts pre-test probabilities in patients and applies the SHAP approach to interpret its decision-making mechanism. This text-based model is expected to have wide applicability.

## Data Availability

The original contributions presented in the study are included in the article/Supplementary Material, further inquiries can be directed to the corresponding author.
