# Supplementary material for: An explainable machine learning model for predicting chronic coronary disease and identifying valuable text features
Source: Front Cardiovasc Med. 2025 Sep 22;12:1559831. doi: 10.3389/fcvm.2025.1559831 (PMC12497772; doi:10.3389/fcvm.2025.1559831)
Supplement: Supplementary file 2 [file Datasheet1.docx]

Optimal hyperparameters of KNN:

n_neighbors: 7

weights: distance

Optimal hyperparameters of Logistic Regression:

C: 1

penalty: l2

Optimal hyperparameters of RandomForestClassifier:

max_depth: None

n_estimators: 200
